# Working conditions for healthcare workers at a Swedish university hospital infectious disease department during the COVID-19 pandemic: barriers and facilitators to maintaining employee wellbeing

**DOI:** 10.3389/fpsyg.2023.1183084

**Published:** 2023-05-18

**Authors:** Malin Veje, Karolina Linden, Verena Sengpiel, Ylva Carlsson, Ingibjörg H. Jonsdottir, Alessio Degl’Innocenti, Linda Ahlstrom, Helle Wijk, Magnus Akerstrom

**Affiliations:** ^1^Department of Infectious Diseases, Sahlgrenska University Hospital, Region Västra Götaland, Gothenburg, Sweden; ^2^Institute of Biomedicine, Department of Infectious Diseases, Sahlgrenska Academy, University of Gothenburg, Gothenburg, Sweden; ^3^Institute of Health and Care Sciences, Sahlgrenska Academy, University of Gothenburg, Gothenburg, Sweden; ^4^Department of Obstetrics and Gynecology, Sahlgrenska University Hospital, Region Västra Götaland, Gothenburg, Sweden; ^5^Center of Perinatal Medicine and Health, Institute of Clinical Sciences, University of Gothenburg, Gothenburg, Sweden; ^6^Institute of Stress Medicine, Region Västra Götaland, Gothenburg, Sweden; ^7^School of Public Health and Community Medicine, Institute of Medicine, Sahlgrenska Academy, University of Gothenburg, Gothenburg, Sweden; ^8^Center for Ethics, Law, and Mental Health (CELAM), Department of Psychiatry and Neurochemistry, Institute of Neuroscience and Physiology, Sahlgrenska Academy, University of Gothenburg, Gothenburg, Sweden; ^9^Gothia Forum for Clinical Trials, Sahlgrenska University Hospital, Region Västra Götaland, Gothenburg, Sweden; ^10^Department of Orthopedics, Sahlgrenska University Hospital, Region Västra Götaland, Gothenburg, Sweden; ^11^Department of Quality Strategies, Sahlgrenska University Hospital, Region Västra Götaland, Gothenburg, Sweden

**Keywords:** COVID-19 pandemic, working conditions, employee wellbeing, healthcare organizations, healthcare workers (HCW), infectious disease departments

## Abstract

**Background:**

Healthcare workers (HCWs) at infectious disease departments have held the frontline during the COVID-19 pandemic. This study aimed to identify barriers and facilitators to maintaining the employees’ wellbeing that may be used to increase preparedness for future pandemics within ID Departments.

**Methods:**

In September 2020, a web-based survey on demographics and work environment was distributed to all HCWs at the Infectious Disease Department at Sahlgrenska University Hospital. Results were compared with a pre-COVID-19 survey from October 2019. A quantitative analysis of the overall effects of the pandemic on the working conditions of HCWs was conducted; in addition, a qualitative content analysis of open-ended responses was performed.

**Results:**

In total, 222 and 149 HCWs completed the pre-COVID-19 and COVID-19 surveys (84 and 54% response rate), respectively. Overall, we found significant changes regarding increased workload, lack of emotional support in stressful work situations, and inability to recover after shifts. These factors correlated both with younger age and concern of becoming infected. The open-ended answers (*n* = 103, 69%) revealed five generic categories (*Workload*; *Organizational support*; *Worry and ethical stress*; *Capability*; and *Cooperation and unity*) with a total of 14 identified factors representing plausible individual and organizational-level barriers or facilitators to sustained employee wellbeing.

**Conclusion:**

Younger HCWs as well as those expressing worries about contracting the infection were found to be particularly affected during the COVID-19 pandemic and these groups may require additional support in future outbreaks. Factors both increasing and decreasing the pandemic-induced negative health consequences for HCWs were identified; this knowledge may be utilized in the future.

## Introduction

Since the beginning of the COVID-19 pandemic, healthcare workers (HCWs) at hospital infectious disease (ID) departments have constituted an important part of the frontline. ID departments have primary responsibility for treatment and isolation of patients with contagious diseases, as well as for providing guidance to other departments regarding infection control and prevention. Compared with neighboring countries, Sweden had many COVID-19 patients early in the pandemic, at a time when the knowledge base about COVID-19 was limited. This exacerbated the burden on hospital beds and HCWs. The number of ICU beds in Sweden is lower than in other European countries, which means that more severely ill patients need to be treated at regular wards (Public Health Agency of Sweden, n.d.; Bauer et al., 2020).

During the pandemic, ID HCWs have experienced unprecedented changes in work environment and tasks. Early in the pandemic, there were reports of high COVID-19 infection rates and mortality among HCWs (Zhan et al., 2020). Previous studies on HCWs during the pandemic, focusing on health effects rather than effects on the HCWs’ working conditions, have shown a negative impact on mental health, especially among frontline workers caring directly for COVID-19 patients (Ruiz-Fernández et al., 2020; Sanghera et al., 2020; Alexiou et al., 2021; Chen et al., 2021; Moradi et al., 2021; Lohela-Karlsson and Condén, 2022; Peccoralo et al., 2022). Interestingly, a large German survey on hospital HCWs showed higher levels of depressive and anxiety symptoms during the first wave of the pandemic than before, but lower levels compared to those in the general population during the pandemic (Morawa et al., 2021).

Reports on HCWs working specifically in ID departments are very scarce. A Korean questionnaire study performed during the spring of 2020 found high levels of burnout and depression among 115 ID physicians (Park et al., 2020). Similarly, a Chinese study with 2,299 participants found that the frontline medical staff, including 213 ID HCWs, were twice more likely than the administrative staff at the same hospital to suffer anxiety and depression (Lu et al., 2020). A recently published Dutch study noticed a deterioration of psychosocial working conditions for frontline workers during the pandemic, but does not state the percentage of the included participants working in an ID department (van Elk et al., 2023). A reduced job satisfaction compared with before COVID-19 was noted in a multinational survey among nurses, of which 118 ID department employees, including in Sweden (Makowicz et al., 2022). A recently published Swedish report on healthcare managers’ work situation during the first COVID-19 pandemic wave found that managers of departments with high COVID-19 exposure reported more difficulties with decision-making authority in addition to a higher workload and less time for recovery, compared with managers with lower COVID-19 exposure (Björk et al., 2022).

While it is now clear that HCW distress has been increased by the COVID-19 pandemic, there is still limited knowledge about which factors increase susceptibility to and which factors can mitigate negative health consequences for HCWs during outbreaks (Pollock et al., 2020). Recently, a mixed-methods study on midwives in the United Kingdom showed changes in working practices resulting in increased job demands during the pandemic. It also suggested job resources that could help mitigate the negative health consequences on the midwives, such as ensuring adequate access to personal protective equipment as well as the importance of being valued and listened to in the workplace (McGrory et al., 2022).

Infectious disease departments have a crucial role in infectious disease outbreaks. To enable them to continue to care for patients during future pandemics, it is important to investigate how the COVID-19 pandemic affected working conditions specifically for ID HCWs.

The objective of the current study was to identify barriers and facilitators to maintaining the ID department employees’ wellbeing, which may be used to increase preparedness for future pandemics. This was done by investigating how the work environment for HCWs at the ID department was affected during the first wave of the COVID-19 pandemic in Sweden.

## Materials and methods

### Study setting

The present study was performed at Sahlgrenska University Hospital, one of the major university hospitals in northern Europe, with approximately 17,000 employees providing care for about 700,000 inhabitants of the Gothenburg region and specialized care for the 1.7 million inhabitants of the region of Västra Götaland in western Sweden.

The study has focused on the HCWs employed at the ID Department. The Department has 62 hospital beds, including four intensive care beds. During the pandemic, several organizational changes were made: The intensive care beds were managed by on-site ICU specialists, instead of by ID specialists in conjunction with ICU doctors on call. Meetings were kept to a minimum, administrative staff who were able to work from home were instructed to do so, and all employees were obliged to stay home and get tested for COVID-19 even with discrete symptoms of infection among themselves or family members. Several nurses from other, non-COVID-19 departments came to work at the ID Department and therefore needed to be trained and supervised by the regular staff. In the beginning of the pandemic, relatives of deceased patients were not allowed to visit the morgue for a final goodbye.

To limit the effect of the pandemic on the HCWs’ wellbeing, the employer offered three types of preventive measures to the HCWs: scheduled meetings for collegial support; information on work environment and COVID-19; and individual debriefing sessions with the occupational health services (OHS). At the time of the study, personal protective equipment (PPE) was provided, but the COVID-19 vaccine was not yet available.

### Study design

In September 2020, a web-based survey (the COVID-19 survey, designed to be completed in 10–20 min, and previously described in detail; Jonsdottir et al., 2021), was distributed to all employees at the hospital, including the ID Department. In the survey, respondents were asked to recall how they experienced their situation during the first pandemic wave in the spring of 2020. The survey contained demographic questions including age, gender, and professional role, as well as 11 single-item questions regarding work environment conditions addressing job demands, support, job motivation, and recovery (Table 1), based on the job demands-resources model. This model assumes that all job characteristics can be classified as either a job demand (i.e., physical, social, or organizational aspects of the job that are associated with physiological or psychological costs) or a job resource (i.e., positively valued physical, social, or organizational aspects that help staff achieve work goals), and that there needs to be a balance between job demands and job resources. Too high job demands in relation to available job resources have the potential to lead to health impairment, while sufficient job resources in relation to job demands can motivate workers and generate engagement, personal growth, learning, and development (Bakker and Demerouti, 2007).

**Table 1:** Percentage of health care workers (HCWs) at the Infectious Disease (ID) Department reporting negative responses (i.e., *disagree* or *strongly disagree*) on the work environment items in the pre-COVID-19 and the COVID-19 survey, respectively, and odds ratios for *disagree* or *strongly disagree* during, versus before, the COVID-19 pandemic.

Survey item	2019		2020		Odds ratio	
	*n*	%	*n*	%	OR	95% CI
I know what is expected of me in my work.	221	1.8	147	19.7	13.3	4.6–38.8
The quantity of my work seems reasonable.	221	12.7	148	51.4	7.3	4.4–12.1
I am able to take part in planning how my work is to be performed.	221	5.9	145	39.3	10.4	5.4–19.9
In my work, my skills and abilities are used in the right way.	221	5.9	146	13.7	2.5	1.2–5.3
My line manager helps me prioritize my work tasks as needed.	221	13.6	146	21.9	1.8	1.0–3.1
I can get help and support if emotionally stressful situations arise in my work.	219	6.4	145	17.9	3.2	1.6–6.4
I have scope for recovery during the work session through breaks and/ or rests.	221	10.0	147	51.7	9.7	5.6–16.7
I look forward to going to work.	220	2.3	147	27.2	16.1	6.2–41.9
I can set thoughts about work aside in my free time.	220	11.8	147	60.5	11.5	6.8–19.4
I have enough energy to do other things after the end of my shift.	220	21.4	147	63.9	6.5	4.1–10.4
I feel rested and recovered after a few days off.	222	9.9	147	57.1	12.1	7.0–21.0

CI, Wald’s confidence interval; *n*, number of respondents; OR, odds ratio for answering disagree or strongly disagree during, versus before, the COVID-19 pandemic.

All items were presented as statements with five response alternatives (*strongly agree*, *agree*, *neither agree nor disagree*, *disagree*, and *strongly disagree*). The demographic and work environment-related items had previously been examined in October 2019 within the hospital’s regular systematic work environment management, offering a pre-pandemic measurement (pre-COVID-19 survey). In addition, the COVID-19 survey contained pandemic-specific items including questions regarding worries about becoming infected with COVID-19, access to PPE, and support from the employer, as well as an open-ended item: “*Which positive and negative effects have you experienced during the first COVID-19 wave in the spring of 2020?*” Since the surveys were initiated, developed, and distributed by the employer, using their own digital systems, data from the two measurements could only be matched on a department level and not on a unit or individual level.

### Ethics approval and consent to participate

This study was conducted in accordance with the Declaration of Helsinki and approved by the Swedish Ethical Review Authority (ref. 2020-04771, date of approval October 31, 2020) for studies involving humans. Informed consent to participate was obtained from all study subjects.

The study was performed as a collaboration project with COPE Staff, a Swedish multicenter study aiming to investigate the psychosocial work environment and experiences of caring for pregnant and newborn patients during the COVID-19 pandemic.^1^

### Statistical analyses

The demographic data (age, gender, and professional role), as well as data on COVID-19 specific items, and on the proportion of respondents reporting negative responses (i.e., *disagree* or *strongly disagree*) for the 11 work environment items, is presented as number and percentage.

The impact of the pandemic on HCWs’ working conditions was assessed by calculating the odds ratio and 95% confidence intervals (CIs) for reporting negative responses on the respective work environment item, during the COVID-19 pandemic versus before the COVID-19 pandemic, using logistic regression analysis.

Potential barriers and facilitators to maintaining employee wellbeing were assessed by including interaction terms between COVID-19 status (pre-COVID-19 vs. COVID-19) and age, gender, professional role, and worry about becoming infected, respectively, in the logistic regression models and/or by assessing the association between the work environment conditions and these factors for the COVID-19 survey only using logistic regression analyses according to above. To keep the number of statistical tests low, when investigating these potential barriers and facilitators, we focused on six working environment conditions that were considered *a priori* important for HCWs in the ID Department. These conditions included job demands (clarity in expectations; quantitative work demands) and job resources (emotional support; ability to utilize skills and competence in the right way), as well as job motivation and recovery.

Data analysis was performed using SAS, version 9.4 (SAS Institute, Cary, NC, United States). The significance level was set at α = 0.05, and all tests were two-tailed.

### Qualitative analysis of open-ended questions

The answers were coded and grouped into generic categories with identified factors representing barriers and facilitators to maintaining the employees’ wellbeing, according to content analysis as described by Elo and Kyngäs (2008). In a second step, the identified factors were stratified into barriers or facilitators of employee wellbeing, according to the job demands-resources model (Bakker and Demerouti, 2007), and grouped according to whether they worked on an individual or an organizational level (Dahlgren and Whitehead, 2007).

## Results

Altogether 264 HCWs employed at the ID Department were eligible for participation in the COVID-19 survey, 149 of whom completed the survey (54%). Of these, 103 (69%) provided an answer to the open-ended question. The pre-COVID-19 measurement from 2019 yielded a response rate of 84% (275 HCWs were eligible for participation in the pre-COVID-19 survey).

Background characteristics and results of COVID-19-specific items are presented in Table 2. A total of 83% (*n* = 123) of the respondents of the COVID-19 survey were women. The majority (73%) were registered nurses or assistant nurses (*n* = 72 and *n* = 35, respectively), and 14% (*n* = 21) were physicians. The percentage of different age, sex, and professional roles was similar in the pre-COVID-19 survey (*p* = n.s.).

**Table 2:** Professional role, age, and gender among health care workers (HCWs) responding to the pre-COVID-19 and the COVID-19 survey, and responses on the COVID-19-specific items in the COVID-19 survey.

	Pre-COVID-19 survey	COVID-19 survey
Number of respondents, *n*	222	149
Professional role, *i* (%)		
Physicians	37 (17)	21 (14)
Registered nurses	99 (45)	72 (49)
Assistant nurses	58 (26)	35 (24)
Administrative personnel	20 (9)	9 (6)
Other^1^	8 (4)	9 (6)
Age, *n* (%)		
<29 years	52 (23)	34 (23)
30–39 years	62 (28)	36 (24)
40–49 years	42 (19)	32 (21)
50–59 years	35 (16)	23 (15)
>60 years	29 (13)	24 (16)
Gender, *n* (%)		
Women	183 (82)	123 (83)
Men	30 (14)	26 (17)
Other/do not want to reply	9 (4)	0 (0)
Caring for COVID-19-infected patients, *n* (%)		
Yes		134 (91)
No		14 (9)
Strong worry about becoming infected, *n* (%)		
Many times per day		11 (8)
Daily		16 (11)
Occasionally		30 (21)
Rarely		49 (34)
Never		40 (27)
Sufficient access to PPE when caring for COVID-19-infected patients, *n* (%)		
Always or most often		124 (85)
Often		5 (3)
Occasionally		0 (0)
Rarely		1 (1)
Rarely or never		0 (0)
Not involved in COVID-19 patient care		16 (11)
Making use of the support provided by the employer, *n* (%)		
Scheduled collegial support		41 (28)
Information on work environment and COVID-19		115 (78)
Debriefing sessions with the OHS		56 (38)

1 Other professional roles included managers, welfare officers, etc.

OHS, occupational health services; PPE, personal protective equipment.

Most HCWs had been involved in direct COVID-19 patient care (*n* = 134; 91%), had been working at their normal department (*n* = 121; 81%; data not shown), and always or mostly had access to adequate PPE (*n* = 124; 85%).

### Impact of the pandemic on health care workers’ working conditions

For all the 11 work environment items, a larger proportion of the respondents in the COVID-19 survey (range 13.7–63.9%) compared to the pre-COVID-19 measurement (1.8–21.4%) reported a negative response (*strongly disagree* or *disagree*), reflecting impaired working conditions during the pandemic. In the pre-COVID-19 survey, only one item out of the 11 had >20% negative responses, whereas in the COVID-19 survey, impaired working conditions with >20% negative responses were reported from the ID HCWs for eight of the 11 items (Table 1).

Odds ratios for reporting a negative response regarding working conditions in the COVID-19 survey versus the pre-COVID-19 survey ranged between 1.8 (95% CI 1.0–3.1) and 16.1 (95% CI 6.2–41.9), with slightly higher odds ratios for items representing job demands, job motivation, and recovery compared to job resources (Table 1).

### Factors affecting the impact of the pandemic on health care workers’ working conditions

When investigating factors affecting the impact of the pandemic on ID HCWs’ working conditions, an overall interaction effect was seen between COVID-19 status (pre- and COVID-19 survey) and age, with a larger proportion of negative responses among younger HCWs. No interaction effects were seen between COVID-19 status and gender or professional role.

**Figure 1:**
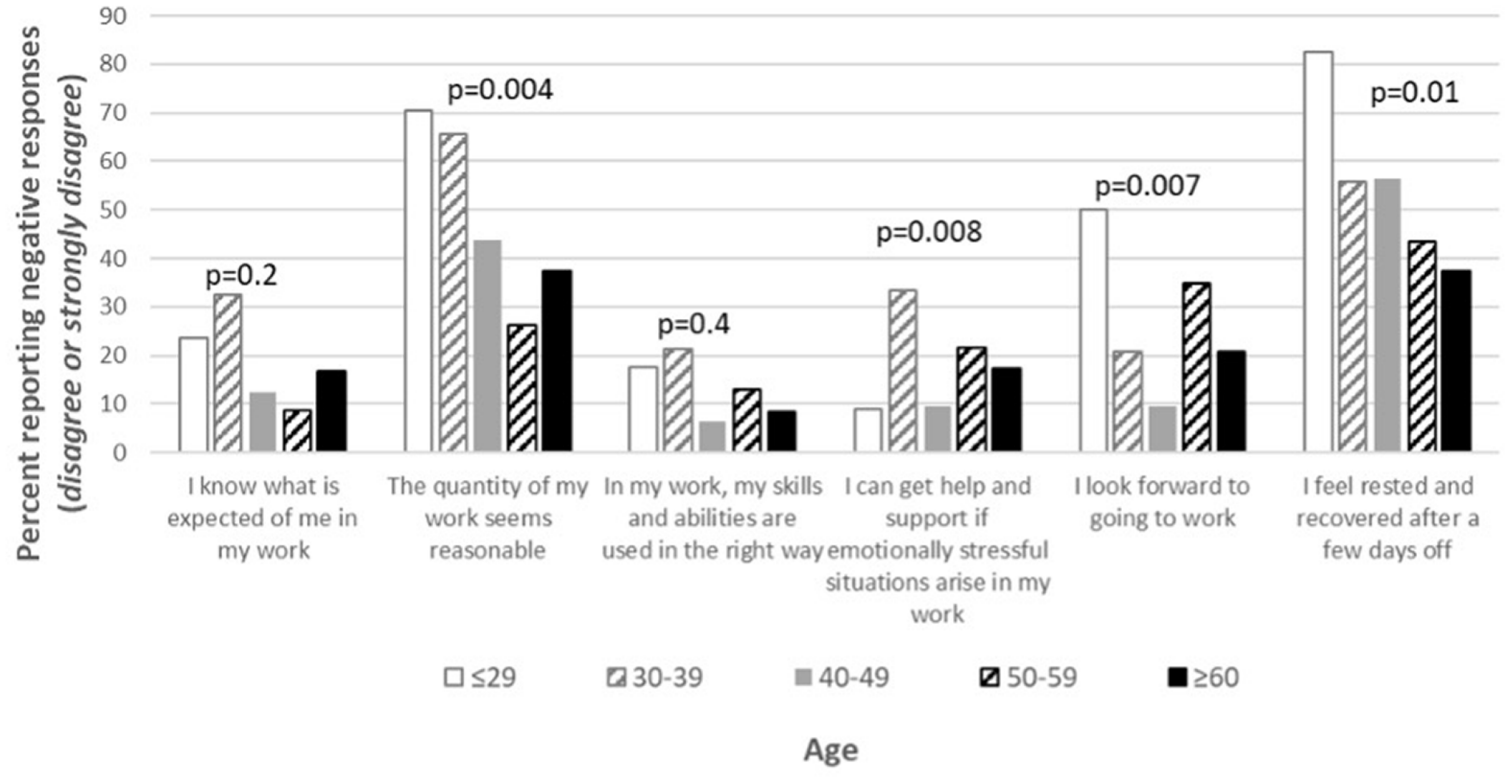
Percentage of health care workers (HCWs) at the infectious disease (ID) Department reporting negative responses (i.e., *disagree* or *strongly disagree*), stratified into age and strong worry about being infected, on selected work environment items in the COVID-19 survey.

In the analysis of the effect of age and strong worry about becoming infected on the six selected work environment conditions in the COVID-19 survey, younger age, and frequent strong worry about becoming infected were associated with a higher proportion of HCWs reporting adverse working conditions. Those HCWs who, on a daily basis, experienced a strong worry about becoming infected reported a higher percentage of negative responses compared to HCWs who did not worry about becoming infected (Figure 1).

Lastly, we investigated the effect of support provided to the ID HCWs by the employer. Between 28 and 78% of HCWs used different types of support provided during the first wave of the pandemic (Table 1). Health care workers who participated in the three different provided support activities were more likely to report a lack of emotional support [odds ratio between 2.5 (95% CI 1.0–6.3) and 12.7 (95% CI 1.7–96.8)] compared to HCWs who did not use the provided support (Table 3). Furthermore, HCWs who attended debriefing sessions with the OHS were less likely to report negative responses regarding job motivation and recovery compared to HCWs who did not use this type of support [odds ratio 0.4 (95% CI 0.2–0.9); Table 3].

**Table 3:** Odds ratios for reporting negative responses (i.e., *disagree* or *strongly disagree*) on selected work environment items in the COVID-19 survey if using, versus not using, the support provided by the employer.

Survey item	*n*	Scheduled collegial support		Information on work environment and COVID-19		Debriefing sessions with the OHS	
		OR	95% CI	OR	95% CI	OR	95% CI
I know what is expected of me in my work.	147	1.0	0.4–2.5	1.9	0.7–4.6	0.6	0.3–1.3
The quantity of my work seems reasonable.	148	0.8	0.4–1.6	2.7	1.2–6.2	0.5	0.3–1.1
In my work, my skills and abilities are used in the right way.	146	1.7	0.5–5.3	2.3	0.8–6.4	0.7	0.3–1.9
I can get help and support if emotionally stressful situations arise in my work.	145	12.7	1.7–96.8	2.5	1.0–6.3	3.2	1.1–8.9
I look forward to going to work.	147	1.2	0.5–2.8	1.1	0.4–2.5	0.4	0.2–0.9
I feel rested and recovered after a few days off.	147	0.8	0.4–1.7	1.9	0.8–4.3	0.4	0.2–0.9

CI, Wald’s confidence interval; *n*, number of respondents; OHS, occupational health services; OR, odds ratio for answering disagree or strongly disagree if making use, versus not making use, of the support provided by the employer.

### Qualitative analysis of the health care workers’ perceptions of working during the pandemic

**Figure 2:**
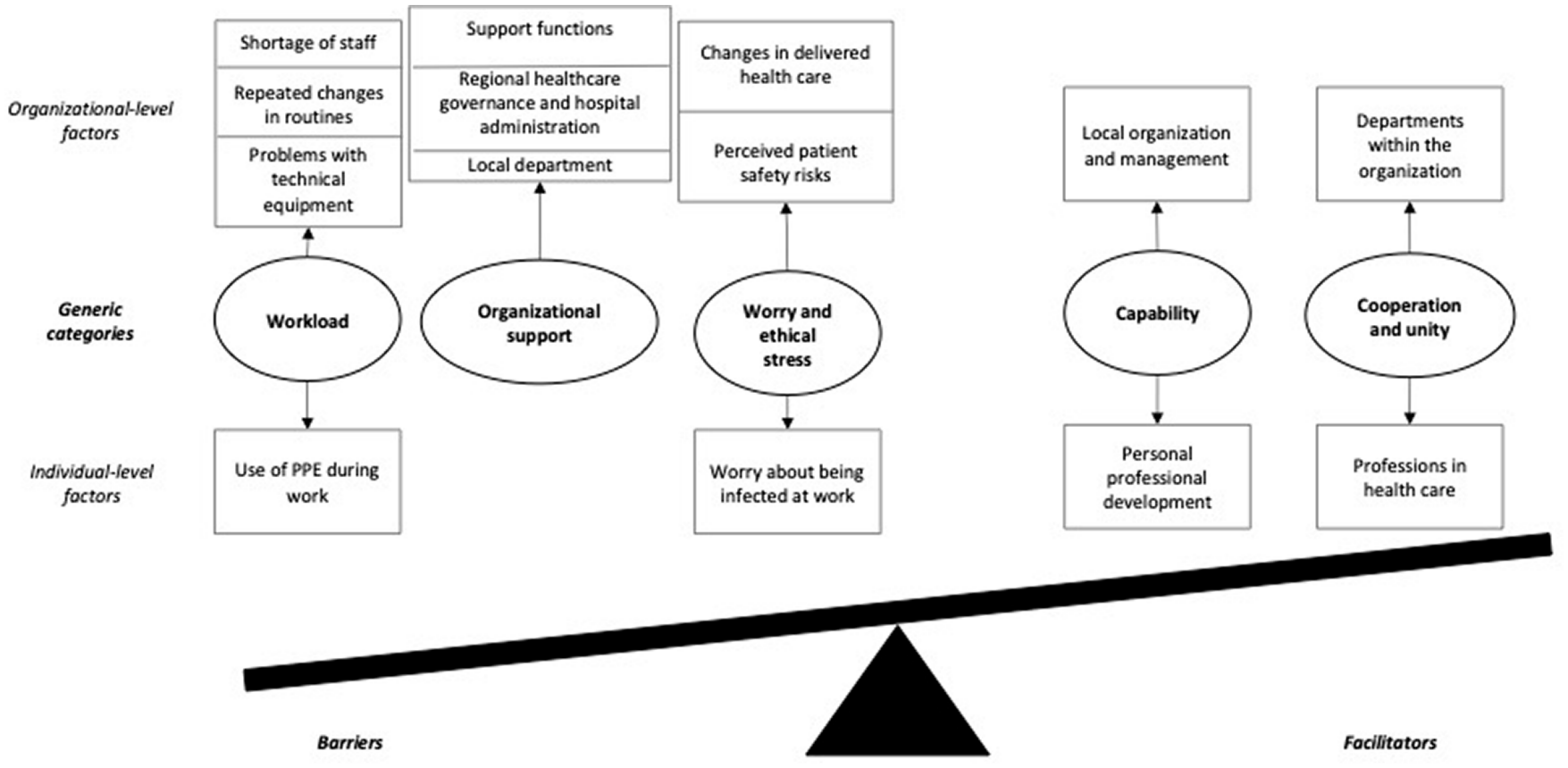
Generic categories with identified factors, stratified into barriers to and facilitators for occupational health, according to the job demands-resources model. PPE, personal protective equipment.

The qualitative analysis resulted in five generic categories (*Workload*; *Organizational support*; *Worry and ethical stress*; *Capability*; and *Cooperation and unity*) related to 14 identified factors representing plausible barriers and facilitators to sustained employee wellbeing during the COVID-19 pandemic. These generic categories and factors included both barriers and facilitators on an individual and organizational level (Figure 2). The generic categories are described in detail below.

#### Workload

A general increase in workload was reported. The work situation was described as chaotic and stressful. There was a lot of time pressure; working overtime was more common than before, routines were changed very often, and there was a perceived lack of recovery after the shift. The HCWs experienced difficulties in maintaining a good work–life balance. Problems with technical equipment were mentioned. There was a widespread sense of exhaustion when working long hours in full PPE, which was exacerbated by the heat and perceived insufficient ventilation in the wards. Many employees described a shortage of staff, resulting in a feeling of being stuck in the patient rooms for hours without being replaced by a colleague. The fact that many HCWs were transferred to the ID Department and needed training added to the workload of the regular ID staff. The cooperation between employees with different professions did not always run smoothly. New research projects that were initiated during the COVID-19 pandemic also added to the workload of the personnel, whereas normal employment training programs were postponed. Some respondents described a sense of being on duty 24/7, partly because of the large media coverage and questions from friends and family.

#### Organizational support

Some respondents voiced their frustration with how the support systems at the hospital were managed. For example, the IT support was described as slow or dysfunctional. Frustration about long lead times at the laboratory was particularly prominent in the beginning of the pandemic. Furthermore, support and information from the Human Resources Department were perceived as insufficient. Initially, there was much frustration about the cooperation with the morgue, where the staff at the morgue were perceived as slow and were described as not making adequate decisions, for example when declining to receive visits from relatives of deceased patients.

Some respondents stated that there was a lack of information from the ID Department’s management, both in general and regarding new work tasks and PPE routines. Moreover, information was shared on a “need-to-know” basis, but there were no clear guidelines about who needed to know what, which led to frustration among the HCWs. Some respondents felt that routines were not always thoroughly elaborated. It was mentioned that the Department’s management should have made better use of existing competence among the staff. Planning of work schedules and staff was regarded as insufficient. There were also complaints about a lack of professional emotional support, especially for staff working nights and weekends.

A perceived general lack of preparedness for the pandemic was mentioned. Lack of direct communication between the Department and the hospital’s top management was also mentioned. There was dissatisfaction over the fact that personnel from the ID Department were not represented in the top level of management at the hospital during the pandemic. Some respondents mentioned that resources were distributed unequally between different departments, which led to frustration among the staff. The perceived deficits in hospital administration also affected the HCWs on a personal level, for example, vacations were canceled or reduced at short notice. Lack of economic compensation was perceived by some respondents as unfair and led to a sense of feeling undervalued by the top hospital management. Further, extra financial compensation awarded to employees working with COVID-19 patients was changed or withdrawn at short notice. The lack of hospital beds and understaffing, a known problem even before the pandemic, was exacerbated due to COVID-19. Some respondents also mentioned a lack of trust in upper hospital management and stated that they perceived a general lack of centralization regarding major decisions.

#### Worry and ethical stress

Concerns over patient safety were voiced. Patient transport between different departments was considered unsafe. Because of the large number of COVID-19 patients, there was a shortage of some drugs, and as a result, new medications had to be used. Insufficient instructions regarding these new drugs, new work tasks, and new equipment led to a fear of reduced patient safety. The magnitude of the workload and the long shifts of the ID HCWs contributed to these concerns.

A fear of contracting the infection was stated by several HCWs. For some respondents, difficulties preparing for a constantly changing work situation led to a general sense of insecurity. Lack of medical knowledge regarding COVID-19 caused worries. Work was perceived as emotionally challenging. In addition, some respondents worried that the hospital would run out of PPE. The uncertainty about the HCWs’ annual summer leave also contributed to raised worries for their own health.

Several situations leading to ethical stress were mentioned. A lack of holistic perspective was perceived, where because of the high workload, HCWs had to focus only on emergency care and not provide the emotional support that they were used to providing. Some patient meetings were very difficult and a sense of insufficiency regarding contact with patients’ relatives was described. The fact that, in the beginning of the pandemic, patients’ relatives were not allowed to visit the morgue caused distress among the HCWs.

#### Capability

A high proportion of respondents praised the adaptability in the organization of the ID Department. The clear leadership, exercised both at unit and at department level, was highly appreciated. Decisions were perceived as measured, which led to a sense of calm and security. Some respondents appreciated that new guidelines were immediately put into effect. Access to PPE was considered adequate. The Department was generally perceived as well functioning. Crisis management was offered by the unit managers. The fact that the organization made extra resources available was appreciated. Given the patient safety risks mentioned above, the drug shortage situation was perceived as well handled, with clear instructions given. COVID-19 testing of staff was organized within the Department, which was valued by the respondents.

Several respondents emphasized the joy of learning new professional skills. Research initiatives were likewise appreciated by some. The staff felt a sense of fulfillment in working with tasks that they were trained for and in finally testing their skills in a real pandemic. Some mentioned a feeling of confidence regarding their personal professional skills. The staff valued the care relationships formed with COVID-19 patients. The continuous real-life process improvement was appreciated and led to a sense of building preparedness for future epidemics and serious events.

#### Cooperation and unity

The cooperation between different departments within the same hospital organization, i.e., the Internal Medicine, ICU, and ID Departments, was perceived as excellent by some respondents. In addition, great help was received from various other departments in the hospital, by contributing additional medical staff. The distribution of patients between different departments was deemed fair. It was helpful that new ICU patient beds were created, both at the ID Department and elsewhere. It was also greatly appreciated that the support from the Human Resources Department improved over time and that hospital transport and cleaning services extended their services.

Several respondents praised the interprofessional cooperation between different professions. There was a widespread feeling of unity within the work groups. A general, very strong sense that everybody was willing to work hard and support each other was emphasized, as well as the joy of going to work. Emotional support between colleagues was perceived as strong. Furthermore, widespread appreciation from the general public was gratefully acknowledged. Some respondents mentioned getting a boost out of being in the center of events.

## Discussion

The results from our study show that HCWs at the ID Department experienced both increased job demands and a decrease in job resources during the first wave of the COVID-19 pandemic, compared to before the pandemic. Overall, the negative effect was larger for the job demands than for the job resources. A similar deterioration in working conditions during the pandemic has previously been described among hospital HCWs in a Spanish multicenter study (Gálvez-Herrer et al., 2022). In our study, no differences were seen between different types of HCWs, which is in contrast with previous research (Chatzittofis et al., 2021). However, being younger, and having frequent worry about contracting COVID-19 were factors associated with perceiving more adverse working conditions compared to others. The same tendency has previously been described for other HCWs including those in, but not restricted to, ID departments during both the COVID-19 and other infectious disease outbreaks (Kisely et al., 2020; Bueno-Notivol et al., 2021; Peccoralo et al., 2022), indicating that specific groups may need extra attention during extraordinary situations in health care.

Lessons from previous epidemics, especially severe acute respiratory syndrome (SARS) and Middle East respiratory syndrome (MERS), have pointed at the importance of adopting a qualitative approach to better understand the needs of, and find the best support for, frontline HCWs during pandemics (Maunder et al., 2006; Cabarkapa et al., 2020; Billings et al., 2021). When analyzing the open-ended item, both negative factors, plausibly acting as barriers to promoting employee wellbeing, and positive factors judged as facilitators, were identified. The negative factors experienced during the pandemic, which might partly explain the perceived deterioration in the HCWs’ working conditions, can be summarized as an increased workload, a perceived lack of organizational support, worries about becoming infected, and ethical stress from not being able to perform patient care as usual. More specifically, the HCWs described challenges related to: use of PPE and technical equipment; repeated changes in routines; and staff shortages; as well as perceived lack of support from internal support functions, their own department, the hospital’s top management, and the regional healthcare government; perceived patient risks; and worry about becoming infected. Similar negative effects on the working conditions were also described in a qualitative study including predominantly HCWs at ID wards in another Swedish county (Rücker et al., 2021), where focus group interviews with 51 participants revealed two main themes: “Concerns about the risk of infection and transmission to others” and “Transition from chaos to managing in a new and challenging work situation.”

Among the positive factors, increased capability, and cooperation and unity were judged as facilitators that might partly counterbalance the plausible effect of a poor work environment on employees’ health and wellbeing. The increased capability was experienced as personal professional development in the ID Department, but also as development of the department and management, where staff were able to follow protocols and routines which, previously, they had only been using in training scenarios. The increased cooperation and unity were found both between different departments at the hospital and between different professions and individuals in the ID Department. Interestingly, the recent Swedish study from Lohela-Karlsson et al., found negative health consequences in HCWs who were involved directly in COVID-19 patient care compared to HCWs who were not, but the consequences were less grave than in countries with a higher COVID-19 burden during the first pandemic wave (Lohela-Karlsson and Condén, 2022).

The discussed barriers and facilitators could be used to identify effective preventive measures in the context of the challenges at hand—measures that have the potential of increasing the resilience of a healthcare organization (McFillen et al., 2013). Consequently, for the ID Department, securing access to internal support functions, including the IT Department, increasing the vertical communication and trust within the organization, increasing the communication concerning changes in routines and patient safety, securing enough support for less experienced HCWs, and addressing the HCWs’ concerns of getting infected may all be important preventive measures to increase the resilience of the Department for future critical situations. In addition, measures that promote the positive findings concerning the increased capability and increased cooperation and unity could also improve working conditions at the ID Department even during normal operations.

The qualitative analysis also demonstrated that factors underlying the identified barriers and facilitators that may affect HCWs’ wellbeing were found at both an individual and an organizational level, highlighting the need for a multi-level approach when improving HCWs’ working conditions (Hasson, 2005; Martin et al., 2016). Therefore, to successfully implement preventive measures at the ID Department based on the above, measures aiming to improve the organizational preconditions, such as securing sufficient resources for managers to enable their active involvement in the daily operations, and further developing the psychosocial safety climate, need to be included (Demartini et al., 2020).

Our results also indicate an imbalance between the job demands and resources, possibly with a resultant decrease in job motivation and possibility for recovery during the COVID-19 pandemic, compared to before the pandemic. Such effects on job motivation and possibility for recovery have previously been seen to be a result of high work demands and may lead to adverse effects on HCWs’ wellbeing and health, as has been described for other frontline workers during the pandemic (Chersich et al., 2020; Salazar de Pablo et al., 2020; van Elk et al., 2023). However, more distal health effects, such as sickness absence and employee turnover, could not be investigated as part of this study because the surveys were designed and distributed by the employer and restricted to items mainly concerning the HCWs’ working conditions.

To reduce potential negative effects on the HCWs’ wellbeing during the COVID-19 pandemic, the employer offered three types of support at an individual and/or group level (scheduled sessions for collegial support, information on work environment and COVID-19, and debriefing sessions with the OHS), which were used by 30–80% of the HCWs at the ID Department. When comparing perceived working conditions between HCWs who used these support measures with those who did not, results revealed that HCWs using the support to a larger extent perceived a lack of emotional support compared to others. One speculation is that HCWs lacking emotional support were more likely to seek, or be referred to, these support measures, thus indicating that the measure targeted the right group. These findings further highlight the need for a multi-level approach when improving working conditions. Health care workers attending debriefing sessions facilitated by the OHS experienced a somewhat smaller negative effect on job motivation and recovery compared to those not attending the sessions, indicating that debriefing sessions may potentially play an important part in reducing adverse effects on employee wellbeing during the acute phase of a pandemic.

### Strengths and limitations

One strength of this study is the mixed method design including a pre- and post-COVID-19 measurement of perceived working conditions and qualitative data on HCWs’ experiences of working during the first phase of the COVID-19 pandemic. Our study focuses particularly on HCWs at the ID Department, who not only possess the skills to treat severely ill patients with contagious diseases, but also play an important role in the healthcare organization as experts during a pandemic, and therefore need to maintain a functioning service during extraordinary events.

A limitation of the study is the use of aggregated data, which enabled us to compare the pre- and post-measurements on a group level, but not to follow the responses of individual participants over time, nor make adjustments for employee turnover. Another limitation was the somewhat low number of items in the survey, which prevented us from investigating a potential impact on more distal health effects. Moreover, although the selected items represented the job demands-resources model, there may be other effects on HCWs’ working conditions, which were not investigated in this study.

## Conclusion

This mixed method study with pre-COVID-19 and COVID-19 measurements has pinpointed both increased job demands and a decrease in job resources for HCWs at a large ID Department during the pandemic. Factors both increasing and decreasing the pandemic-induced negative health consequences for HCWs were identified, which may be useful knowledge for future disease outbreaks. An increased workload, a perceived lack of organizational support, concerns about becoming infected, and ethical stress from not being able to perform patient care as usual were found to be barriers to employee wellbeing. Meanwhile, increased capability and cooperation and unity were found to be facilitators of employees’ health and wellbeing. In addition, younger HCWs and HCWs with a strong concern about contracting the infection may require additional support in future outbreaks. By ensuring emotional, managerial, and peer support, especially directed at these groups, we may be able to lessen the burden on frontline HCWs in future pandemics.

## Data availability statement

The raw data supporting the conclusions of this article will be made available by the authors, without undue reservation.

## Ethics statement

The studies involving human participants were reviewed and approved by the Swedish Ethical Review Authority (ref. 2020-04771, date of approval October 31, 2020) for studies involving humans. The patients/participants provided their written informed consent to participate in this study.

## Author contributions

MV, KL, VS, YC, IJ, AD’I, LA, HW, and MA: conceptualization. MV, KL, and MA: methodology, formal analysis, and original draft preparation. YC, KL, VS, MV, MA, IJ, AD’I, LA, and HW: review and editing. KL and YC: funding acquisition. All authors contributed to the article and approved the submitted version.

## Funding

This study was funded by FORMAS (ref. No. 2020-02767) and grants from the Swedish state under the ALF agreement between the Swedish government and the county councils (YC; ALFGBG-75710). The funders had no role in study design, data collection and analysis, decision to publish, or preparation of the manuscript.

## Conflict of interest

The authors declare that the research was conducted in the absence of any commercial or financial relationships that could be construed as a potential conflict of interest.

## Publisher’s note

All claims expressed in this article are solely those of the authors and do not necessarily represent those of their affiliated organizations, or those of the publisher, the editors and the reviewers. Any product that may be evaluated in this article, or claim that may be made by its manufacturer, is not guaranteed or endorsed by the publisher.

## Footnotes

^1^www.snaks.se/cope-staff
